# Free Fatty Acids Activate Renin-Angiotensin System in 3T3-L1 Adipocytes through Nuclear Factor-kappa B Pathway

**DOI:** 10.1155/2016/1587594

**Published:** 2015-12-31

**Authors:** Jia Sun, Jinhua Luo, Yuting Ruan, Liangchang Xiu, Bimei Fang, Hua Zhang, Ming Wang, Hong Chen

**Affiliations:** ^1^Department of Endocrinology, Zhujiang Hospital, Southern Medical University, Guangzhou, China; ^2^Department of Geratology, The Affiliated Hospital of Guangdong Medical College, Guangdong Medical College, Zhanjiang, Guangdong, China; ^3^Department of Epidemiology and Medical Statistics, School of Public Health, Guangdong Medical College, Dongguan, Guangdong, China; ^4^Second Clinical School of Medicine, Southern Medical University, Guangzhou, China; ^5^Nephrology Center of Integrated Traditional Chinese and Western Medicine, Zhujiang Hospital, Southern Medical University, Guangzhou, China

## Abstract

The activity of a local renin-angiotensin system (RAS) in the adipose tissue is closely associated with obesity-related diseases. However, the mechanism of RAS activation in adipose tissue is still unknown. In the current study, we found that palmitic acid (PA), one kind of free fatty acid, induced the activity of RAS in 3T3-L1 adipocytes. In the presence of fetuin A (Fet A), PA upregulated the expression of angiotensinogen (AGT) and angiotensin type 1 receptor (AT_1_R) and stimulated the secretion of angiotensin II (ANG II) in 3T3-L1 adipocytes. Moreover, the activation of RAS in 3T3-L1 adipocytes was blocked when we blocked Toll-like receptor 4 (TLR4) signaling pathway using TAK242 or NF-*κ*B signaling pathway using BAY117082. Together, our results have identified critical molecular mechanisms linking PA/TLR4/NF-*κ*B signaling pathway to the activity of the local renin-angiotensin system in adipose tissue.

## 1. Introduction

Activation of the renin-angiotensin system (RAS) is instrumental in regulating blood pressure and fluid balance. RAS activation is also associated with impaired differentiation of preadipocytes [1] and increased lipolysis and enhanced oxidative stress and inflammatory response [2–8]. Defects in the system are associated with obesity, type 2 diabetes, and cardiovascular diseases. RAS are found in a number of tissues, including kidneys, heart, and nervous and immune systems. Components of RAS, including renin, angiotensinogen (AGT), angiotensin-converting enzyme (ACE), and angiotensins I, II, and III (ANG I, ANG II, and ANG III), have also been found in adipose tissue [9, 10]. It is well established that free fatty acids (FFAs) are activators of RAS in leukocytes [11, 12]. However, whether FFAs play a role in the activation of RAS in adipocytes is unclear. It has been shown that the levels of FFAs originating from lipolysis in adipocytes are significantly increased in peripheral circulation as well as local tissues in obese humans and animals [13, 14]. It has been hypothesized that RAS could regulate adipocyte differentiation through Ang II and the adipocyte AT_1_R in mice [15]. Therefore, FFAs may directly regulate RAS activation in adipose tissue which might be a trigging mechanism of glucose and lipid metabolism disorder and obesity-related diseases. FFA components such as palmitic acid (PA) and lauric acid can bind to Toll-like receptor 4 (TLR4) with the assistance of the endogenous ligand, fetuin A (Fet A), thus mediating the activation of TLR4 and NF-*κ*B pathways and leading to the inflammatory cascade [14]. TLR4 is a member of the family of Toll-like receptors (TLRs), which can activate mitogen-activated protein kinase and nuclear factor *κ*B (NF-*κ*B) to regulate inflammatory and immune responses after binding to ligands [16]. Moreover, a recent study has demonstrated that active TLR4 can induce the activation of RAS in hepatocytes [17] and cardiac muscle cells [18]. Therefore, we hypothesize that palmitic acid (PA) triggers the TLR4 signaling pathway, leading to RAS activation in adipocytes.

## 2. Material and Methods 

### 2.1. Reagents

We purchased 3T3-L1 preadipocyte line from ATCC (CL-173); Dulbecco's Modified Eagle Medium (DMEM) with 25 mM D-glucose from HyClone (USA); Dexamethasone (DXM), Isobutylmethylxanthine (IBMX), 4% paraformaldehyde, Oil Red O, Irbesartan, and Captopril from Sigma (Sigma-Aldrich, St. Louis, USA); dimethyl sulfoxide (DMSO) from Invitrogen (USA); Ang II Enzyme-linked immunosorbent assay kit from Cusabio (Wuhan, China); Anti-TLR4 antibody from Abnova (Taiwan, China); anti-AGT antibody from Merck (Merck Millipore, Darmstadt, Germany); anti-AT_1_R antibody and anti-GAPDH antibody from Santa Cruz (CA, USA); Anti-*α*-tubulin antibody from Cell Signaling Technology (Beverly, MA, USA); and horseradish peroxidase-linked goat-anti-rabbit antibody from KPL (Gaithersburg, MD, USA).

### 2.2. Cell Culture, Differentiation, and Identification

3T3-L1 preadipocytes were subcultured with 25 mM D-glucose DMEM. Two days after confluence, the cells were differentiated to adipocytes using the same medium containing 10% fetal bovine serum (FBS, HyClone), supplemented with 10 *μ*g/mL insulin, 1 *μ*M DXM, and 0.5 mM IBMX. This medium was replaced with a fresh medium containing insulin 48 hours later, after which the medium was replaced every other day. Approximately 90%–95% of cells differentiated into mature adipocytes on days 8–10 of culture, which were used in the experiments. After the 3T3-L1 preadipocytes were completely differentiated, 4% paraformaldehyde was added to the culture dish and maintained for 10 min, stained with Oil Red O staining for 30 min, followed by stain extraction and observation using a microscope.

### 2.3. Preparation of Palmitic Acid

Palmitic acid (PA) and bovine serum albumin (BSA) were purchased from Sigma (Sigma-Aldrich, St. Louis, USA). PA were dissolved completely in 0.1 M NaOH at 70°C and then complexed with 9.5 mL 10% BSA at 55°C for 10 min such that a final PA concentration of 5 mM was achieved. Stock solutions were stored at 4°C after filtration or diluted with DMEM to one-tenth (500 *μ*M PA) or one-twentieth (250 *μ*M PA) that were prepared fresh before experiments.

### 2.4. Enzyme-Linked Immunosorbent Assay

3T3-L1 adipocytes were treated, respectively, with DMEM, DMEM + 0.1% BSA, DMEM + 0.1% BSA + 10 *μ*g/mL Fet A, or DMEM + 0.1% BSA + 500 *μ*M PA for 3 hours or treated in 500 *μ*M PA + Fet A condition for 1, 3, or 5 hours. In other experiments, 3T3-L1 adipocytes were treated with 250 *μ*M PA + 10 *μ*g/mL Fet A or 500 *μ*M PA + 10 *μ*g/mL Fet A in the same vehicle, containing DMEM and 0.1% BSA, for 3 hours. The supernatants were collected and the concentrations of ANG II were determined by double antibody sandwich method, using Ang II* Enzyme-linked immunosorbent assay* kit. The OD values were measured by a microplate reader, and then ANG II concentration was calculated.

### 2.5. Quantitative RT-PCR

Amplification and detection of RNA were performed in an ABI Prism 7300 Sequence Detection System using SYBR Green (Applied Biosystems, Foster City, CA, USA), according to the manufacturer's instructions. Primers for quantitative RT-PCR were designed based on sequences from the GenBank, as follows. The relative mRNA expression level was calculated using the comparative expression level 2^−ΔΔCT^ method:

TLR4: F: 5′-GCATCATCTTCATTGTCCTTGA-3′,

R: 5′-CTTGTTCTTCCTCTGCTGTTTG-3′;

AGT: F: 5′-CCTTCCATCTCCTTTACCACAA-3′,

R: 5′-GCAGGGTCTTCTCATTCACAG-3′;

AT_1_R: F: 5′-TGCCATGCCCATAACCATCTG-3′,

R: 5′-CGTGCTCATTTTCGTAGACAGG-3′;

GAPDH: F: 5′-GGAAGCCCATCACCATCTT-3′,

R: 5′-GGTTCACACCCATCACAAACAT- 3′.

### 2.6. Western Blotting Analysis

Protein extract was separated on a 15% SDS-polyacrylamide gel and electrophoretically transferred onto a PDVF membrane (Millipore, Etten-Leur, The Netherlands). Membranes were blocked overnight with 5% nonfat dried milk and incubated for 2 h after washing with TBST (10 mM Tris, pH 8.0, 150 mM NaCl, and 0.1% Tween 20), and the membranes were incubated for 1 h with horseradish peroxidase-linked goat-anti-rabbit antibody. The membranes were washed again with TBST, and the proteins were visualized using ECL chemiluminescence.

### 2.7. Immunofluorescence Double Staining

3T3-L1 adipocytes were treated with 500 *μ*M PA after pretreatment with two kinds of RAS blocking agents, respectively; one is an angiotensin receptor blocking agent (ARB), Irbesartan, with 10 *μ*M concentration; the other is an inhibitor of ACE (ACEI), Captopril, with 10 *μ*M concentration. Cells were then fixed by paraformaldehyde after the supernatant was removed. FITC + DAPI double staining method was used to detect nuclear translocation of the p65 subunit of NF-*κ*B.

### 2.8. Statistical Analysis

All statistical analyses were performed using SPSS version 16.0 software. Results were presented as means ± SD. Student's *t*-test was used to compare the means between two samples and statistical comparisons of more than two groups were performed using one-way analysis of variance (ANOVA); post hoc tests were performed using LSD test or Tamhane's T2 test. *P* values < 0.05 were considered statistically significant.

## 3. Results

### 3.1. Combined Fet A and PA Upregulated the Expressions of AGT, AT_1_R, and TLR4 and Stimulated the Secretion of ANG II in 3T3-L1 Adipocytes

**Figure 1:**
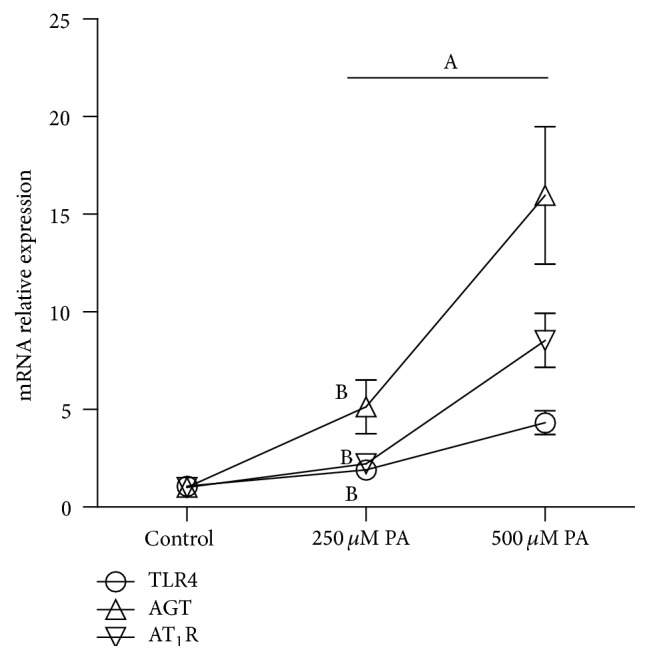
mRNA expression of TLR4, AGT, and AT_1_R in 3T3-L1 adipocytes induced by PA. Data are presented as mean ± SD. (A) *P* < 0.05 versus control, (B) *P* < 0.05 versus 500 *μ*M PA ($\overset{-}{x}\pm s$, *n* = 9).

**Figure 2:**
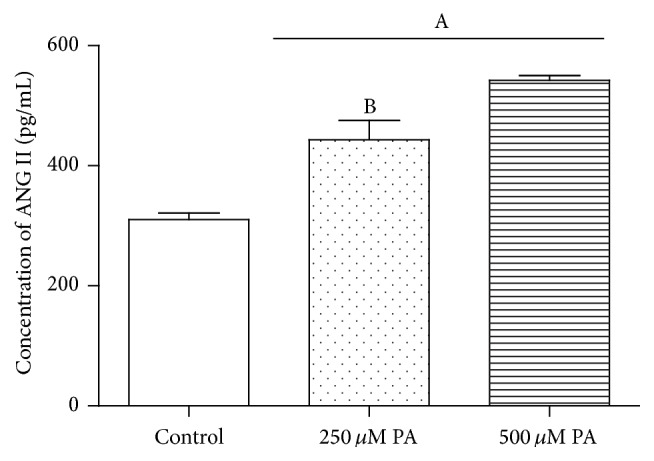
Concentrations of ANG II secreted by 3T3-L1 adipocytes after being treated by different concentrations of PA. Data are presented as mean ± SD. (A) *P* < 0.05 versus control, (B) *P* < 0.05 versus 500 *μ*M PA ($\overset{-}{x}\pm s$, *n* = 6).

**Figure 3:**
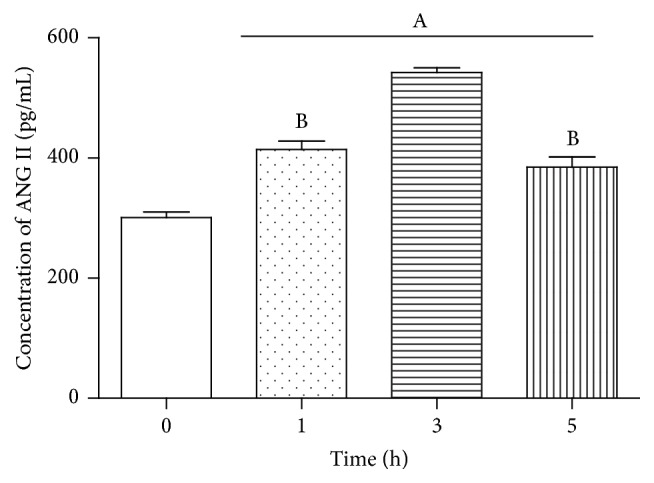
Concentrations of ANG II secreted by 3T3-L1 adipocytes after being treated with PA + Fet A at different time. Data are presented as mean ± SD. (A) *P* < 0.05 versus control, (B) *P* < 0.05 versus 3 h ($\overset{-}{x}\pm s$, *n* = 6).

To investigate whether the involvement of Fet A has an effect on the components of RAS induced by PA in adipocytes, we conducted the following experiments. 3T3-L1 adipocytes were treated with DMEM, DMEM + 0.1% BSA, DMEM + 0.1% BSA + 10 *μ*g/mL Fet A, or DMEM + 0.1% BSA + 500 *μ*M PA for 3 hours, respectively. We found that there were no significant differences in mRNA expressions of AGT, AT_1_R and secretion of ANG II between groups. In particular, treatment group of PA or Fet A alone has no significant effect in mRNA expressions of RAS components (*P* > 0.05) (data not shown). In contrast, when 3T3-L1 adipocytes were treated with DMEM + 0.1% BSA + 250 *μ*M PA + 10 *μ*g/mL Fet A or DMEM + 0.1% BSA + 500 *μ*M PA + 10 *μ*g/mL Fet A for 3 hours, PA increased the mRNA expressions of TLR4, AGT, and AT_1_R (Figure 1) and the secretion of ANG II (Figure 2). We also test the optimal Fet A and PA treatment time for the secretion of ANG II. We found that the optimal time was 3 hours and time longer than 3 hours caused unspecific effect (Figure 3).

### 3.2. Combination of Fet A + PA Totally Lost the Effect on the Expressions of AGT and AT_1_R in the 3T3-L1 Adipocytes When Blocking TLR4 Beforehand

**Figure 4:**
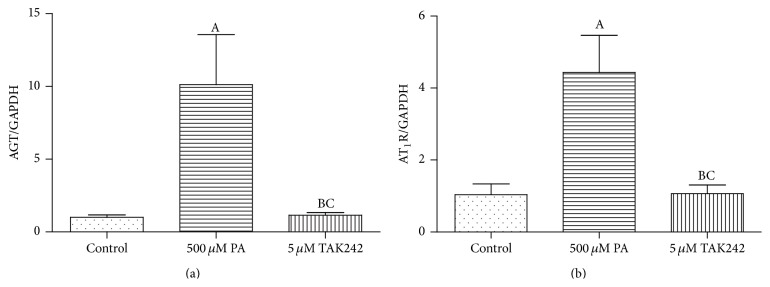
TAK242 pretreatment prevented upregulation of AGT and AT_1_R mRNA expressions. Data are presented as mean ± SD. (A) *P* < 0.05 versus control, (B) *P* < 0.05 versus PA group, and (C) *P* > 0.05 versus control ($\overset{-}{x}\pm s$, *n* = 9).

To investigate whether TLR4 is the medium of PA affecting RAS component expression, we pretreated 3T3-L1 adipocytes with 5 *μ*M TLR4 inhibitor-TAK242 for 1 hour and then treated with DMEM + 0.1% BSA or DMEM + 0.1% BSA + 500 *μ*M PA + 10 *μ*g/mL Fet for 3 hours, respectively. Compared with the Fet A + PA alone group, TLR4 inhibitor-TAK242 completely blocked the expressions of AGT and AT_1_R in the mRNA level (Figure 4) and protein level (Figure 6).

### 3.3. Combination of Fet A + PA Only Partly Lost the Effect on Expressions of AGT and AT_1_R in the 3T3-L1 Adipocytes When Blocking NF-*κ*B Beforehand

**Figure 5:**
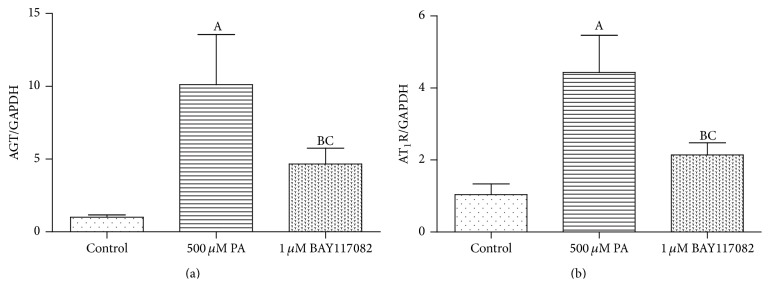
BAY117082 pretreatment partly prevented upregulation of AGT and AT_1_R mRNA expressions. Data are presented as mean ± SD. (A) *P* < 0.05 versus control, (B) *P* < 0.05 versus PA group, and (C) *P* > 0.05 versus control ($\overset{-}{x}\pm s$, *n* = 9).

**Figure 6:**
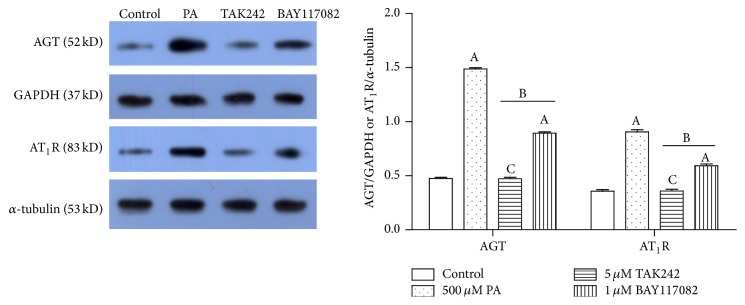
Effects of PA with or without TLR4/NF-*κ*B inhibitors on AGT and AT_1_R protein expression in the 3T3-L1 adipocytes. Data are presented as mean ± SD. (A) *P* < 0.05 versus control, (B) *P* < 0.05 versus 500 *μ*M PA, and (C) *P* > 0.05 versus control ($\overset{-}{x}\pm s$, *n* = 3).

To investigate whether NF-*κ*B is the medium of PA affecting the expression of RAS components, we pretreated 3T3-L1 adipocytes with 1 *μ*M NF-*κ*B inhibitor-BAY117082 for 1 hour and then treated with DMEM + 0.1% BSA or DMEM + 0.1% BSA + 500 *μ*M PA + 10 *μ*g/mL Fet for 3 hours, respectively. Compared with the Fet A + PA alone group, NF-*κ*B inhibitor-BAY117082 only partly blocked the expressions of AGT and AT_1_R in the mRNA level (Figure 5) and protein level (Figure 6).

### 3.4. Combination of Fet A + PA Enabled the Translocation of p65 Subunit of NF-*κ*B to the Nucleus in the 3T3-L1 Adipocytes, and the Effect Was Blocked by RAS Inhibitors

**Figure 7:**
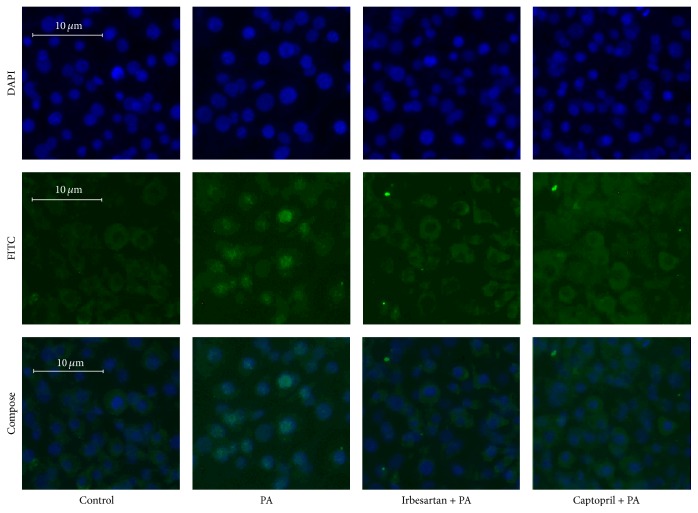
Effect of PA with or without Irbesartan or Captopril pretreatment on NF-*κ*B p65 subunit translocation in 3T3-L1 adipocytes. DAPI: DAPI staining of nucleus; FITC: FITC staining of NF-*κ*B p65 subunit; Compose: composite images of DAPI and FITC.

3T3-L1 adipocytes were treated with DMEM + 0.1% BSA (group 1) or DMEM + 0.1% BSA + 500 *μ*M PA + 10 *μ*g/mL Fet A (group 2) for 3 hours or pretreated with DMEM + 10 *μ*M Irbesartan for 1 hour followed by DMEM + 0.1% BSA + 500 *μ*M PA + 10 *μ*g/mL Fet A for 3 hours (group 3) or pretreated with DMEM + 10 *μ*M Captopril for 1 hour followed by DMEM + 0.1% BSA + 500 *μ*M PA + 10 *μ*g/mL Fet A (group 4) for 3 hours. The intensity of green fluorescence of FITC in the nucleus of group 2 was stronger, and the cytoplasm of group 2 was weaker than the other 3 groups. The intensity of green fluorescence of FITC in the nucleus and cytoplasm was almost similar in the control (group 1), Irbesartan pretreatment (group 3), and Captopril pretreatment (group 4) groups (Figure 7).

In summary, we found that PA upregulated the expressions of AGT and AT_1_R in both gene and protein level, as well as the gene expression of TLR4 in 3T3-L1 adipocytes. The PA-induced enhancement of AGT resulted in increasing secretion of ANG II, which is believed to be a crucial early step in the development of adipocytes inflammation. Moreover, we found that RAS activation mediated by PA in adipocytes needs to act through TLR4 signaling pathway but not entirely to be dependent on TLR4 downstream NF-*κ*B pathway.

## 4. Discussion

AGT, the precursor of ANG II, is mainly expressed in adipocytes [19], which is an important component of RAS in adipose tissue. AGT gets converted to ANG II after being catalyzed by the components of RAS-renin and ACE and the increased generation of ANG II responses to upregulation of AGT expression [4]. Therefore, AGT expression is the symbol of local RAS activation [2, 8, 10]. In addition to the elevation of AGT expression, local RAS activation is often accompanied by the increased expression of AT_1_R, which is the major ANG II receptor expressed in adipocytes, mediating a series of pathophysiological effects [20]. In current study, we show that PA with Fet A, a liver secretory glycoprotein which exists in blood circulation [21], upregulated the expression of AGT and AT_1_R and the secretion ANG II in 3T3-L1 adipocytes. Our results confirm that the activation of RAS in the adipose tissues is mediated by PA.

Next, we try to identify the signaling pathways of activation of local adipose RAS mediated by PA infusion. We found that PA with Fet A induced TLR4 activation in 3T3-L1 adipocytes. To further confirm PA-mediated activation of the adipose RAS through TLR4 signaling pathways, TLR4 blocker TAK242 was used to block TLR4 signal pathway before addition of PA and Fet A. We found that the TLR4 blocker completely prevented elevating expressions of AGT, ANG II, and AT_1_R, confirming that activation of adipose RAS depends on the TLR4 signaling pathway.

NF-*κ*B, a nuclear transcription factor [22], also upregulated AGT and AT_1_R, the RAS components, in rat vascular smooth muscle cells [23] and preglomerular vascular smooth muscle cells [23]. Previous studies also found that Ang II could activate NF-*κ*B and its downstream inflammatory pathways [20]. To examine whether NF-*κ*B is involved in the activation of adipocyte RAS, we tested the expressions of AGT, ANGII, and AT_1_R in PA with Fet A induced RAS activation after using NF-*κ*B inhibitors. We found that PA with Fet A still caused a marginal increase in the expressions of AGT, ANGII, and AT_1_R with the pretreatment of NF-*κ*B inhibitors. Together, our results suggest that the activation of adipose RAS completely depends on the TLR4 signaling pathway but only partly depends on NF-*κ*B although NF-*κ*B can be activated by TLR4 signaling pathways.

To further vindicate the PA/TLR4/NF-*κ*B signaling pathway, we examined the effect of PA with Fet A on the nuclear translocation of NF-*κ*B. We found that PA with Fet A stimulated the nuclear translocation of NF-*κ*B P65, resulting in eventually increasing NF-*κ*B activity in 3T3-L1 adipocytes. However, this effect is diminished or partly prevented by ACEI or ARB pretreatment, further indicating that PA-induced activation of adipose RAS is correlated with NF-*κ*B activation.

**Figure 8:**
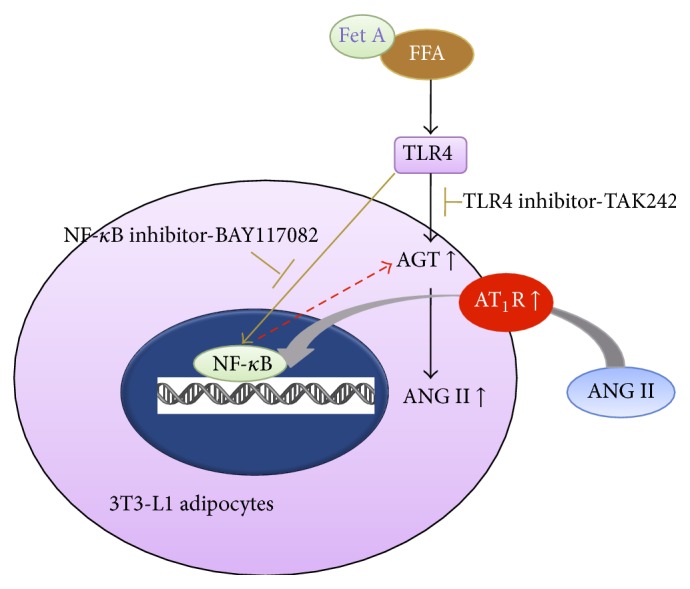
Mechanism of RAS activation induced by FFA (PA) in 3T3-L1 adipocytes.

We present a schematic diagram to explain the pathways of activation of adipose RAS induced by PA/Fed A through TLR4/NF-*κ*B signaling pathway in 3T3-L1 adipocytes (Figure 8). When combining act with Fed A, PA enhances TLR4/NF-*κ*B activity, subsequently upregulating the gene and protein expression of AGT and AT_1_R in adipocytes. PA-induced enhancement of AGT results in increasing secretion of ANG II, which is a crucial early step in the development of adipocytes inflammation. Moreover, the adipose RAS activation mediated by PA/TLR4 is not entirely dependent on NF-*κ*B.

Our findings identify a potential mechanism involved in the pathogenesis of obesity-related diseases.
